# The Impact of Breastfeeding on Health Outcomes for Infants Diagnosed with Neonatal Abstinence Syndrome: A Review

**DOI:** 10.7759/cureus.3061

**Published:** 2018-07-28

**Authors:** Danwei Wu, Camille Carre

**Affiliations:** 1 College of Medicine, University of Central Florida, Orlando, USA; 2 College of Medicine, University of Central Florida , Orlando, USA

**Keywords:** neonatal abstinence syndrome, breastfeeding, methadone, buprenorphrine, opiod

## Abstract

Neonatal abstinence syndrome (NAS) is a neurologic condition resulting from prenatal exposure to opioids. The sudden cessation of opioids in neonates can lead to withdrawal symptoms affecting the neurologic, respiratory, and gastrointestinal systems. Rising opioid use in the United States has led to an increased incidence of infants born with NAS. Despite the growing incidence of NAS, there is a lack of standardized guidelines for intervention and management. Recent studies suggest that non-pharmacological methods should be used as first-line interventions for the reduction of NAS symptoms. Of the non-pharmacological methods, growing literature suggests that breastfeeding may have the potential to reduce symptom severity and improve outcomes. We searched the PubMed and Medline databases for experimental/quasi-experimental studies published from 1997-2018 regarding outcomes in breastfed versus formula-fed neonates with prenatal exposure to opioids. Seven retrospective studies fulfilling the inclusion criteria were reviewed. Collectively, the studies show a strong correlation between breastfeeding and a reduced length of hospital stay, a decreased severity of NAS presentation, and a decreased necessity of pharmacological interventions in infants diagnosed with NAS. From these findings, we recommend breastfeeding as an integral component of the early management of NAS.

## Introduction and background

Neonatal abstinence syndrome (NAS) refers to a broad array of neurological and developmental symptoms that result from prenatal exposure to opioids and/or other psychoactive substances. Symptoms include irritability, hypertonia, tremors, feeding intolerance, hyperactive bowels, seizures, and respiratory distress [1-3]. The pathophysiology of NAS is not completely understood; it is believed to be due to a sudden cessation of psychoactive substances following birth, leading to a precipitation of withdrawal symptoms [2,4]. Neurotransmitter imbalance, as well as variable levels of opioid receptor expression, is thought to contribute to the underlying pathophysiology of NAS, giving rise to autonomic, sensory, and motor dysregulation [5]. A clinical diagnosis of NAS is made if infants have a history of prenatal exposure to psychoactive substances as well as the corresponding signs and symptoms of withdrawal. The Finnegan scoring system is the most commonly used tool for the assessment of neonatal abstinence syndrome (Table 1). Assessment is performed every three to four hours to monitor the severity of neurologic excitability, gastrointestinal dysfunction, autonomic instability, and respiratory dysregulation. A Finnegan score greater or equal to eight commonly warrants intervention, however, the protocol for treatment greatly differs between hospitals [6].

**Table 1:** Finnegan score The Finnegan score assesses symptoms that are most frequently observed in opiate-exposed infants. The scoring usually occurs every three to four hours after birth. For many institutions, three consecutive scores ≥ 8 leads to the initiation of treatment for withdrawal. However, thresholds for treatment may vary between institutions and treatment protocol is not standardized.

Systems	Signs and Symptoms	Score
Central nervous system	High-pitched cry	2
	Continuous high-pitched cry	3
	Sleeps < 1 hour after feeding	3
	Sleeps < 2 hours after feeding	2
	Sleeps < 3 hours after feeding	1
	Hyperactive Moro reflex	1
	Markedly hyperactive Moro reflex	2
	Mild tremors: disturbed	1
	Moderate to severe tremors: disturbed	2
	Mild tremors: undisturbed	1
	Moderate to severe tremors: undisturbed	2
	Increased muscle tone	1-2
	Excoriation (indicate area)	1-2
	Generalized seizure	8
Metabolic vasomotor/respiratory	Fever ≥ 37.2⁰C (99⁰F)	1
	Frequent yawning (≥ 4 in an interval)	1
	Sweating	1
	Nasal stuffiness	1
	Sneezing (≥ 4 in an interval)	1
	Tachypnea (rate > 60/min)	2
Gastrointestinal	Poor feeding	2
	Vomiting (or regurgitation)	2
	Loose stools	2
	90% of birth weight	2
	Excessive irritability	1-3

In the United States, a rising incidence of opioid abuse has led to a corresponding increase in the incidence of NAS from 1.2 to 5.8 per 1,000 hospital births from the time period between 2000 and 2012 [7]. In addition, admission rates for NAS have risen from seven to 27 cases per 1,000 admissions from the time period between 1999 and 2013. As the incidence of NAS increases, hospital expenditures for the treatment of NAS have increased as well. Between the years of 2000 to 2009, total hospital charges for NAS increased from $190 million to $720 million [8]. Studies show that 58% of infants of mothers receiving methadone maintenance therapy (MMT) and 67% of infants of mothers receiving buprenorphine maintenance therapy (BMT) will develop NAS [9]. To fully understand the burden of the opioid epidemic, it is imperative to understand the social, economic, and health implications associated with the rising incidences of NAS. In addition, research and the standardization of treatment can potentially improve long-term outcomes for infants diagnosed with NAS and reduce health costs.

Current treatments for neonatal abstinence syndrome

Pharmacological intervention is necessary in 27% to 91% of neonates with NAS depending on regional and institutional differences [2,10-11]. While there is no universal guideline for the treatment of NAS, pharmacological intervention is not recommended unless supportive therapy fails and severe symptoms, such as seizures and gastrointestinal distress, persist. Recommended first-line treatment involves non-pharmacological care, such as swaddling, gentle handling, demand feeding, and stimulus reduction [3]. In terms of types of pharmacologic treatments, morphine is the preferred opioid medication for the treatment of severe NAS associated with neonatal opioid exposure due to its short half-life of three to four hours and relatively safe profile [12-13]. Newer adjunct agents, such as phenobarbital and clonidine, can be used in cases of refractory or non-opiate-related NAS [14]. Research is still ongoing on the efficacy of these two adjuncts as potential monotherapy [2].

Breastfeeding and guidelines

Breastfeeding greatly improves health outcomes in infants and is associated with lower rates of infection, childhood metabolic diseases, and post-neonatal infant mortality [15-16]. The American Academy of Pediatrics and American Academy of Family Physician recommends exclusive breastfeeding during the first six months of life and for an additional one year with the incorporation of solid foods unless there are contraindications such as maternal human immunodeficiency virus (HIV), chemotherapy, radiation therapy, and illicit drug/polydrug abuse [15]. Previously, breastfeeding was discouraged for mothers receiving opioid maintenance therapy (OMT) due to the belief that the breast milk may contain levels of opioids harmful to infants and neonates [17]. Studies now show that only low concentrations of methadone are secreted in breast milk (ranging from 21-462 ng/mL) in a dose-independent manner [18-19]. Guidelines endorsed by the American Academy of Pediatrics in 2001 and reaffirmed in 2013 have removed the restriction on breastfeeding for mothers on all dosages of methadone [20-21]. Research now demonstrates that the concentration of opiates in infants breastfed by mothers on OMT is negligible and that there were no neurological sequelae from the exposure of opiates through breast milk [18].

## Review

Methods

We accessed the PubMed and Medline databases and reviewed articles that met our search term criteria. Search terms included neonatal abstinence syndrome, methadone, morphine, buprenorphine, and breastfeeding. We searched only for human studies in English that were published between 1997 and 2018, with a preference for quasi-experimental studies, as no randomized controlled trials were found. To be included, studies must have (1) women who are postpartum on FDA-approved opioid maintenance therapy (OMT), (2) infants diagnosed with neonatal abstinence syndrome (NAS), and (3) assessed the differences in outcomes between breastfed and formula-fed infants. The PubMed and Medline search yielded 58 articles of which seven met the inclusion criteria (Table 2).

**Table 2:** Summary of the impact of breastfeeding status on health outcomes among infants diagnosed with neonatal abstinence syndrome Seven studies matching our search criteria were included in this review. The main parameters evaluated include: (1) length of hospital stay, (2) severity of neonatal abstinence syndrome, and (3) length of required pharmacologic intervention.

Study	Year	Type	Sample size	Length of hospital stay	Severity of neonatal abstinence syndrome	Length of pharmacologic intervention
Abdel-Latif et al. [19]	2006	Retrospective chart review	190	Shorter length of hospital stay in breastfed infants (14.7 ± 14.9) vs formula-fed (19.1 ± 15.0); (P<0.05)	Mean Finnegan score is lower in breastfed infants (4.8 ± 0.14) as compared to formula-fed (5.7 ± 0.18); (95% CI, P <0.05). (95% CI, P<0.05)	Breastfed infants were less likely to require pharmacological intervention 52.9% for breastfed infants vs 79% in formula-fed infants; (P<0.001)
Dryden et al. [22]	2009	Retrospective chart review	450	-	-	Infants that were breastfed for ≥ 72 hours (n=99) while in the hospital for treatment of NAS were less likely to require pharmacological intervention; (OR 0.55, 95%, CI 0.34 - 0.88; P = 0.13)
Isemann et al. [23]	2011	Retrospective chart review	128	Shorter length of hospital stay in breastfed infants (median=12.5) versus formula-fed infants (median = 18.6); (B = -0.03, P = 0.02)	-	-
McQueen et al. [24]	2011	Retrospective chart review	28	-	Mean Finnegan score was lower in predominantly breastfed infants (M = 4.9, SD = 2.9) as compared to combination-fed (M = 6.5, SD = 3.7) and formula-fed groups (M = 6.9, SD = 4.2); Kruskal-Wallis test (H(2) = 43.52; P = 0.0001)	-
Pritham et al. [25]	2012	Retrospective chart review	152	Breastfeeding status predicted shorter length of hospital stay; (B = -.3.23, P = 0.05)	-	-
Welle-Strand et al. [26]	2013	Retrospective chart review/prospective study	124	-	Decreased incidence of severe NAS requiring pharmacological intervention in methadone-exposed newborns that were breastfed (53%) as compared to methadone-exposed newborns that are not breastfed (80%) (P<0.05); No difference in buprenorphine-exposed newborns	Shorter duration of pharmacological treatment needed in breastfed newborns (28.6 ± 19.1 days) versus formula-fed newborns (46.7 days ± 26.3); (P<0.05)
Short et al. [27]	2016	Retrospective cohort review	3,725	Breastfeeding status predicted shorter length of hospital stay; (B = -0.085, P = 0.008)	-

A 2006 retrospective chart review conducted by Abdel-Latif et al. evaluated drug-dependent mother and infant pairs. Infants were either breastfed (n=85) or bottle-fed (n=105) beginning on postnatal day five. All mothers were encouraged to breastfeed unless contraindicated (i.e. positive for HIV or maternal intoxication). The main aim of the study was to assess the effect of breastfeeding on the severity and frequency of NAS in infants with drug-dependent mothers. Infants were monitored using the Finnegan scoring system, starting from the first feed and performed before every feed for the duration of the hospital stay. Pharmacological intervention with morphine commenced if the Finnegan’s score was ≥ 8 on two occasions or was > 10 on one occasion. In cases of refractory symptoms, phenobarbitone was administered. Mother and infants were discharged once the Finnegan score was < 8 for at least two days [19].

Abdel-Latif et al. stratified infants by prematurity, polydrug exposure, and methadone for statistical analysis. In the first nine days of life, the mean Finnegan score was lower in breastfed infants (4.8 ± 0.14) as compared to formula-fed (5.7±0.18) (95% CI, P <0.05). The median time to withdrawal symptoms occurred later than in breastfed infants (M=10 days) as compared to formula-fed (M = 3 days) (P<0.001). In addition, breastfed infants were less likely to require pharmacological intervention (OR=0.35, 95%, CI 0.178-0.711; P=0.003). Total hospital stay was shorter in breastfed infants (14.7 ± 14.9) versus formula-fed (19.1 ± 15.0) (P<0.05). These findings suggest that breastfeeding has possible benefits in ameliorating the symptoms of NAS regardless of gestation and type of drug exposure, with the main outcomes including a decrease in the length of hospital stay and a reduced need for pharmacological intervention. Abdel-Latif et al. also assessed the characteristics of breastfeeding mothers. Breastfeeding mothers were more likely to have antenatal care, less likely for positive polydrug abuse, and less likely to be notified as an at-risk parent despite a history of OMT [19].

A 2009 retrospective study by Dryden et al. evaluated the relationship of breastfeeding and pharmacological intervention in infants with NAS. Recent mothers with a history of drug abuse on methadone maintenance therapy (MMT) and their newborns were included in this retrospective review (n=450). Of the total number of infants, 27.7% initiated breastfeeding while in the hospital and 45.5% of infants received pharmacological treatment for NAS. The study found that infants that were breastfed for ≥ 72 hours (n=99) while in the hospital for treatment of NAS were less likely to require pharmacological treatment (OR=0.55, 95% CI, 0.34 - 0.88; P=0.13) [22].

A 2011 retrospective chart review by Isemann et al. assessed 128 newborn infants treated with at least one dose of methadone for NAS to determine if breastfeeding reduced the length of hospital stay. The chart review cross-referenced pharmacological records of infants treated with NAS and records indicating breastfeeding choice. Fifty-six of the 128 infants were breastfed during the hospitalization. Ingestion of breast milk was associated with an inverse correlation in the length of stay (median 12.5 (3 to 51) vs 18.5 (9 to 43) days, P=0.01), suggesting that breastfed infants had a later onset of NAS, an earlier termination of pharmacological treatment, and reduced need for adjunctive therapy [23].

A 2011 retrospective chart review by McQueen et al. examined 28 term infants exposed to methadone in utero and compared neonatal abstinence scores when predominantly breastfed versus predominantly formula-fed to determine if the feeding method affected severity. Infants who were diagnosed with NAS and exposed to methadone were included in this study. Findings showed that infants who were breastfed (M=7.7, SD=3.5) required less monitoring and lower severity scores than infants who were formula-fed (M=11.4, SD=2.9), suggesting that breastfeeding may help decrease symptom severity (P=0.04) [24].

In a 2012 retrospective chart review conducted by Pritham et al., the neonates of mothers on MMT (n=136) and BMT (n=16) were assessed through a review of the electronic medical record for factors affecting the length of hospital stay for NAS. Only women on MMT or BMT at the research hospital were included. Newborns born prematurely (<28 weeks) were excluded from this research. A multiple regression was calculated to predict the length of hospital stay based on maternal methadone dose, smoking status, benzodiazepine, selective serotonin reuptake inhibitors (SSRIs), alcohol, use of other opioids, and infant feeding method. Multiple regression was statically significant, (F (8,101)=3.93, P=.00, R2=0.24) with effects for methadone dose (P=0.02), benzodiazepine exposure (P=0.00), and feeding method (P=0.05), suggesting that breastfeeding is associated with a shorter hospital stay. The study attempts to control for different maternal and infant characteristics, which may obscure the true effects of breastfeeding and demonstrates that breastfeeding is associated with reduced hospital stay despite other confounding factors [25].

Similar conclusions were made in a 2013 multipart retrospective/prospective chart review by Welle-Strand et al. The study assessed 124 women on OMT treated with methadone or buprenorphine during pregnancy through a questionnaire and review of medical records. The aim was to examine the effects of the rate and duration of breastfeeding in women on OMT on infant outcomes with NAS. Of the 124 women and children, 78 neonates (63%) were exposed to methadone and 46 neonates were exposed to buprenorphine (37%). It was found that among the neonates requiring pharmacological treatment for NAS, breastfed infants required a shorter duration of treatment (n=95; 28.6 ± 19.1 days) as compared to those who were bottle-fed (n=29; 46.7 ± 26.3) (P< 0.05). The authors concluded that breastfed methadone-exposed newborns had a lower incidence of NAS and a shorter duration of pharmacological therapy as compared to non-breastfed methadone-exposed newborns [26].

In the study by Well-Strand et al., data were collected from 18 different hospitals in Norway. Unlike other studies assessing for maternal characteristics, Well-Strand et al. found no significant difference in age, education, parity, polydrug abuse, and neonatal growth between breastfeeding and formula feeding mothers. The authors acknowledge this finding is largely due to the relative homogeneity of Norway as compared to North America [26].

A larger 2016 retrospective cohort study by Short et al. evaluated 3,725 neonates with NAS to assess breastfeeding status and length of hospitalization. Infants with the corresponding NAS international classification of disease (ICD) score were selected. Of the evaluated infants, 44.5% were breastfed at discharge. Breastfeeding was associated with a reduction in hospital length from 10 days for breastfed infants (interquartile range 5-19) to 12 days for non-breastfed infants (interquartile range 5-22). There was an inverse relationship between breastfeeding and length of stay (B=-0.085, P=0.008) that remained significant after adjusting for birth year, hospital, neonatal intensive care admission, mode of delivery, birth weight, infant comorbidities, maternal age, race, and marital status (B=-0.060, P=0.05) [27].

In addition, the Short et al. study also evaluated the relationship between the socioeconomic background of the mothers and breastfeeding status. Breastfeeding mothers had a higher education (44.9% vs 32.6%, P<0.0001), smoked during pregnancy (81.0% vs 70.1%, P<0.0001), were more likely to be married (25.2% vs 16.9%, P<0.0001), and had lower rates of Medicaid utilization (66.6% vs 72.6%, P=0.0001) despite positive history of OMT. These findings corroborate with previous studies indicating socioeconomic determinants as important factors influencing maternal decisions to breastfeed [27].

## Conclusions

Based on the result of these studies, hospitals should adopt practices encouraging breastfeeding in newborns with NAS unless otherwise contraindicated. Breastfeeding is well-tolerated and provides health benefits for both mother and infant. Reducing the symptoms of NAS through non-pharmacologic treatments can reduce both the length of hospital stay and financial cost for mothers. Furthermore, breastfeeding strengthens the mother-infant dyad and contributes to the reduction of NAS duration and severity. Offering women resources and facilities where they can discuss barriers to breastfeeding, as well as access to lactation consultants and other trained professionals, can empower mothers to continue breastfeeding. Therefore, hospital practices should provide breastfeeding education and support as part of early intervention for NAS.
